# Research on Stress Variations During the 4H-SiC Indentation Process

**DOI:** 10.3390/mi17010138

**Published:** 2026-01-22

**Authors:** Wenshan Wang, Shuixing Lin, Yiqing Yu, Nian Duan

**Affiliations:** 1 College of Mechanical Engineering and Automation, Huaqiao University, Xiamen 361021, China; 2 Institute of Manufacturing Engineering, Huaqiao University, Xiamen 361021, China

**Keywords:** 4H-SiC, SPH method, indentation simulation, stress, damage

## Abstract

In order to explore the effect of stress on the damage of 4H-SiC materials, this paper employed single abrasive grain indentation simulation based on the Smoothed-Particle Hydrodynamics (SPH) method, and verified the accuracy of the indentation model through an indentation experiment on a single abrasive grain. The research examined the consequences of varying pressures on the processing of 4H-SiC, including parameters such as the depth of abrasive grain penetration, the stress-affected region, and the initiation and propagation of cracks. Subsequently, mathematical models were developed to characterize stress variations under different pressure conditions. The findings reveal several vital insights: First, a discernible linear relationship exists between the depth of abrasive grain penetration into 4H-SiC and the applied pressure. Second, within a specific pressure range, the stress-affected zone within the workpiece enlarges as the applied pressure increases. However, when cracks form within the workpiece, the dimensions of the stress-affected zone exhibit fluctuations. During the abrasive grain indentation phase, a discernible pattern emerges in the stress distribution within the workpiece.

## 1. Introduction

Silicon Carbide (SiC), as a third-generation semiconductor material, possesses characteristics such as a wide band-gap, high breakdown strength, high thermal conductivity, high elastic modulus, and exceptional chemical inertness. At present, SiC has been widely applied in fields such as satellite communication, integrated circuits, and microchips, among others [1,2]. However, SiC’s high hardness and brittleness result in such problems as crack damage and brittle fracture during the machining process, making fabricating its chips relatively challenging [3,4,5]. The central processing steps for SiC wafers involve slicing, grinding, and polishing.

The grinding process of SiC can be primarily divided into two stages: first, the abrasive particles embed into the SiC wafer under the influence of grinding pressure, and then micro-cutting takes place [6]. The embedding of abrasive particles into the workpiece is a process driven by the collective action of multiple abrasive grains, resulting in stress and damage to the workpiece [7]. Currently, the indentation of a single abrasive grain is the most commonly employed method for investigating the mechanical properties of materials by most scholars. It also serves as the foundation for studying the material removal mechanisms by multiple abrasive grains. SU et al. [8] investigated the machining behavior of 6H-SiC under varying pressures. Pang et al. [9], utilizing crystal plasticity theory, delved into the deformation behavior of single-crystal 6H-SiC during nanoindentation. Matsumoto et al. [10] conducted nanoindentation experiments under diverse loading conditions to scrutinize the damage characteristics of 4H-SiC. In a parallel study, Zhang et al. [11] carried out micro-nanoindentation experiments and employed FEM simulations on 6H-SiC. Their analysis of the indentation results under different loads revealed an increase in crack length and indentation depth as the indentation load increased. At the same time, the hardness and elastic modulus exhibited a decrease with higher loads.

Due to limitations imposed by data acquisition frequency and detection techniques, research on micro-nanoscale indentation processes often employs single-indentation simulation methods. In terms of simulation algorithms, FEM has certain limitations when dealing with large deformations. Molecular dynamics simulations generally use nanoscale calculations, and larger-scale calculations require a lot of time. The ideal scale for the grinding process of 4H-SiC wafers falls within the sub-micron range. SPH simulation is generally above the sub-micron level, and is suitable for studying dynamic responses and large deformations in materials. In recent years, more researchers have been employing SPH methods to simulate the stress and strain in brittle material processing. Liu et al. [12] utilized the Smoothed-Particle Hydrodynamics (SPH) method to simulate the nanoindentation process of SiC, and the obtained results for plastic strain and crack evolution closely matched the experimental findings by Holmquist et al. [13]. Shi et al. [14], employing the SPH method, simulated the single-grit machining behavior of SiC, investigating the microscopic mechanical mechanisms influenced by factors such as indentation speed and angle. Guo et al. [15] used the SPH method to explore the stress distribution during abrasive grit scratching of K9 glass. They concluded that different shapes of abrasive grits induce varying stress distributions in the workpiece, with spherical abrasives having the most profound impact on stress. At present, the existing research on SiC indentation mainly focuses on qualitative analysis of damage and stress change in the indentation results, but does not establish a quantitative model of stress change during indentation. Therefore, the present study utilizes the SPH method for simulating the indentation process of 4H-SiC. The accuracy of the indentation model was confirmed by single abrasive grain indentation experiments. Subsequent simulations are carried out to examine the stress variations within 4H-SiC under varying loading pressures and to elucidate the impact of pressure on indentation depth, material damage, and stress alterations in 4H-SiC.

## 2. Simulation and Validation

### 2.1. SPH Theory

Smoothed-Particle Hydrodynamics (SPH) is an unstructured numerical simulation method based on the Lagrangian formulation, initially pioneered by Lucy, Gingold, Monaghan et al. [16,17,18]. It was initially employed to simulate astrophysical phenomena and has since found applications in dynamic response problems involving material strength and fluid dynamics with significant deformations. Liu et al. [19] provided a detailed derivation of the SPH formula. In the SPH algorithm, parameters such as density, velocity, and energy can be represented as integral interpolation using a series of points, and motion information of the particles can be expressed by kernel estimates of these points. The approximate function *f*(*x*) of particles at this point can be defined as follows:

(1) $$f(x)=\int_{\Omega }f\left(x^{\prime }\right)\;\delta \left(x-x^{\prime }\right)dx^{\prime }$$

where *x* is the coordinate vector of the particle, and $\delta \left(x-x^{\prime }\right)$ is the Dirac function. The Dirac function possesses the following properties:

(2) $$\delta \left(x-x^{\prime }\right)=\left\{\begin{array}{c} 1,x=x^{\prime },\\ 0,x\neq x^{\prime }. \end{array}\right.$$

Due to the necessity of the Dirac function to satisfy the defined conditions in Equation (2), Equation (1) cannot be employed for numerical computations. Typically, in numerical simulations, a smooth function $W\left(x-x^{\prime },h\right)$ is used to replace the Dirac function $\delta \left(x-x^{\prime }\right)$. Consequently, the function *f*(*x*) in Equation (1) can be reformulated as follows:

(3) $$f\left(x\right)=\int_{\Omega }f\left(x^{\prime }\right)\;W\left(x-x^{\prime },h\right)dx^{\prime }$$

where *h* is the characteristic smooth length of the influence region of the smooth function *W*.

It is common practice to select even functions that monotonically decrease and remain greater than or equal to zero as the distance from the particle increases in the smooth functions. Additionally, these smooth functions are required to possess the following characteristics:

(4) $$\int_{\Omega }W\left(x-x^{\prime },h\right)\;dx^{\prime }=1$$

(5) $$W\left(x-x^{\prime }\right)=0,\;\;\left|x-x^{\prime }\right|>\mu h$$

(6) $$\underset{h\to 0}{\lim}W\left(x-x^{\prime },h\right)=\delta \left(x-x^{\prime }\right)$$

where μ determines the effective range of the smooth function, and $\left|x-x^{\prime }\right|>\mu h$ is the support domain of *x*.

Among the many smooth functions, the cubic spline functions are the most widely used in the existing Smoothed-Particle Hydrodynamics (SPH) literature. In this study, cubic spline functions are also employed as the smoothing functions $W\left(x-x^{\prime },h\right)$, so that $W\left(x-x^{\prime },h\right)$ is defined as

(7) $$W\left(x-x^{\prime },h\right)=C\times \left\{\begin{array}{l} \frac{2}{3}-y^{2}+\frac{1}{2}y^{3},0\leq y<1,\\ \frac{1}{6}{\left(2-y\right)}^{3},1\leq y<2,\\ 0,y\geq 2. \end{array}\right.$$

where *C* is a constant, $C=1/h,\;15/7\pi h^{2},\;3/2\pi h^{3}$ in one-dimensional, two-dimensional, and three-dimensional spaces, and *y* is the relative distance between points *x* and $x^{\prime }$.

The SPH algorithm system can be represented by particles with independent masses and occupying separate spaces. The continuous integral representation of the SPH kernel approximation method, as in Equation (1), can be transformed into a discrete form as a summation over all particles, which is defined as follows:

(8) $$f\left(x_{i}\right)=\sum_{j=1}^{N}\frac{m_{j}}{\rho_{j}}f\left(x_{j}\right)W\left(x_{i}-x_{j},h\right)$$

where $m_{j}$ is the mass of particle *j*; $\rho_{j}$ is the density of particle *j*; and *N* is the total number of particles within the support domain.

The gradient of the function $f\left(x_{i}\right)$ is defined as

(9) $$\nabla f\left(x_{i}\right)=\sum_{j=1}^{N}\frac{m_{j}}{\rho_{j}}f\left(x_{j}\right)\nabla W\left(x_{i}-x_{j},h\right)$$

Density, momentum, and energy equations can be defined as [20]

(10) $$\frac{d\rho_{i}}{dt}=\sum_{j=1}^{N}m_{j}\left(v_{i}^{\beta }-v_{j}^{\beta }\right)\frac{\partial W_{ij}}{\partial x_{i}^{\beta }}$$

(11) $$\frac{dv_{i}^{\alpha }}{dt}=\sum_{j=1}^{N}m_{j}\left(\frac{\sigma_{i}^{\alpha \beta }}{\rho_{i}^{2}}+\frac{\sigma_{j}^{\alpha \beta }}{\rho_{j}^{2}}\right)\frac{\partial W_{ij}}{\partial x_{i}^{\beta }}$$

(12) $$\frac{de_{i}}{dt}=\frac{1}{2}\sum_{j=1}^{N}m_{j}\left(\frac{p_{i}}{\rho_{i}^{2}}+\frac{p_{j}}{\rho_{j}^{2}}\right)\left(v_{i}^{\beta }-v_{j}^{\beta }\right)\frac{\partial W_{ij}}{\partial x_{i}^{\beta }}$$

where α, β are the coordinate direction, σ is the total stress tensor, and p is the pressure.

### 2.2. Establishment of the Simulation Model

**Figure 1:**
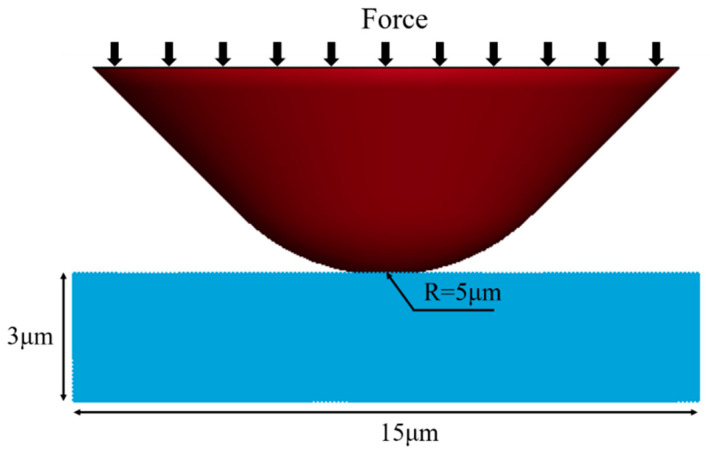
Simulation model.

In the simulation, a consistent system of units is utilized, with μg, μm, and μs as the fundamental units for the simulation. As shown in Figure 1, a three-dimensional single-indentation simulation model is established to conduct the simulation analysis presented in this paper. The 4H-SiC workpiece takes the form of a uniform grid-based rectangular prism with dimensions of 15 μm × 15 μm × 3 μm. The minimum separation between two SPH particles is set at 0.1 μm, resulting in the conversion of the workpiece’s grid into 706,831 SPH particles. Considering the depth of abrasive particle penetration during the machining process, the model simplifies the diamond abrasive particles as conical tips with a radius of 5 μm, comprising 1248 FEM grids. In the simulation procedure, a load denoted as P is progressively applied to the abrasive particle, causing it to indent into the 4H-SiC workpiece gradually.

Because of the high hardness of diamond abrasive particles, minimal deformation and damage occur during the downward loading process. Therefore, the simulations presented in this paper, specifically regarding the use of diamond abrasive particles as rigid bodies and the utilization of the MAT-RIGID material model from LS-DYNA with its associated material properties, are as shown in Table 1 [21]. On the contrary, 4H-SiC, a material characterized by high hardness and brittleness, undergoes crack formation and brittle fracture during the machining process. Thus, the Johnson–Holmquist Ceramics (JH-2) constitutive model is applied. This model is an enhanced iteration built upon the JH-1 model, introducing a gradual softening process before material failure. Furthermore, properties such as strength and damage are dependent on other parameters. The model’s specific parameters are shown in Table 2 [22].

### 2.3. Validation of the Simulation Model

**Figure 2:**
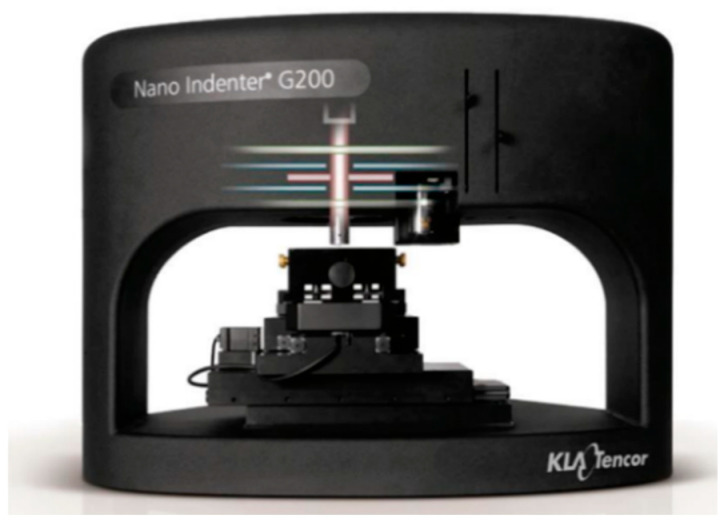
Nano Indenter G200.

In order to ensure the accuracy of the indentation simulation model, comparative experiments with the same parameters need to be conducted for verification. Given that the abrasive particles in the simulation model are set as rigid bodies, it is only necessary to validate the mechanical performance of the 4H-SiC workpiece within the simulation. The experiment utilized double-sided polished 4H-SiC wafers obtained from Beijing Tianke Heda, with a thickness of 330 μm and wafer dimensions of 10 × 10 mm. The initial surface roughness of the samples exhibited an Ra value of less than 1 nm, meeting the precision requirements for the experiment. The experimental apparatus employed was the Nano Indenter G200 (Keysight Technologies, Inc., Santa Rosa, CA, USA), manufactured by KLA-Tencor, a company based in the United States, as shown in Figure 2.

**Figure 3:**
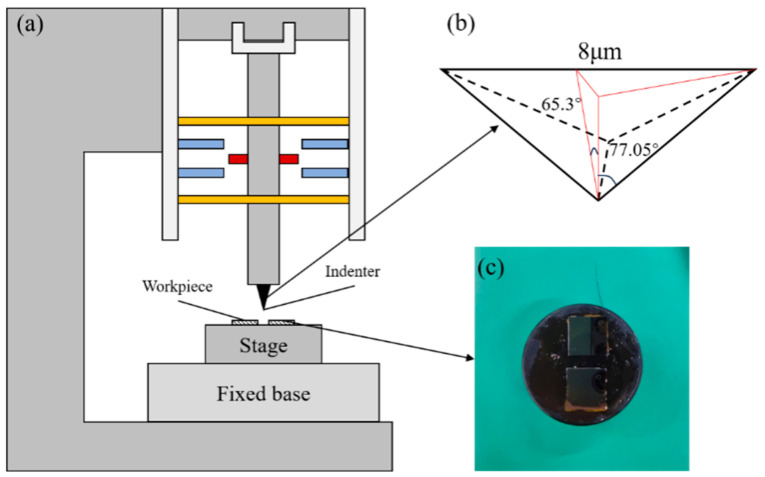
(**a**) Structure of the nanoindentation instrument. (**b**) Berkovich indenter parameters. (**c**) Preparation of 4H-SiC sample.

Figure 3 shows a schematic representation of the experimental setup. A standard Berkovich diamond indenter was used in the experiments, with geometric parameters outlined in Figure 3b. The indenter was securely mounted on the spindle, enabling precise motion control during the indentation process. In order to prevent the sample from falling off during the experiment, the 4H-SiC samples were firmly affixed to the loading stage using paraffin, as shown in Figure 3c. The specific experimental parameters and simulation control parameters are found in Table 3.

**Figure 4:**
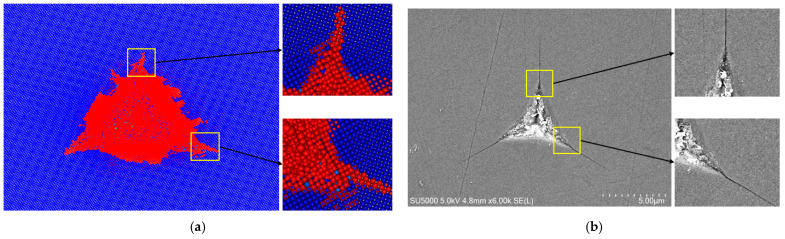
(**a**) Simulated indentation surface topography. (**b**) Experimental indentation surface topography.

Figure 4 shows a comparative illustration of the surface topography when comparing simulated and experimental results for the 4H-SiC indentation. As shown in Figure 4a, the indentation morphology predominantly takes on a triangular shape, with distinct crack propagation evident at the three corners. In the central region of the indentation, the material has been completely destroyed. This phenomenon strongly aligns with the experimental indentation results shown in Figure 4b.

**Figure 5:**
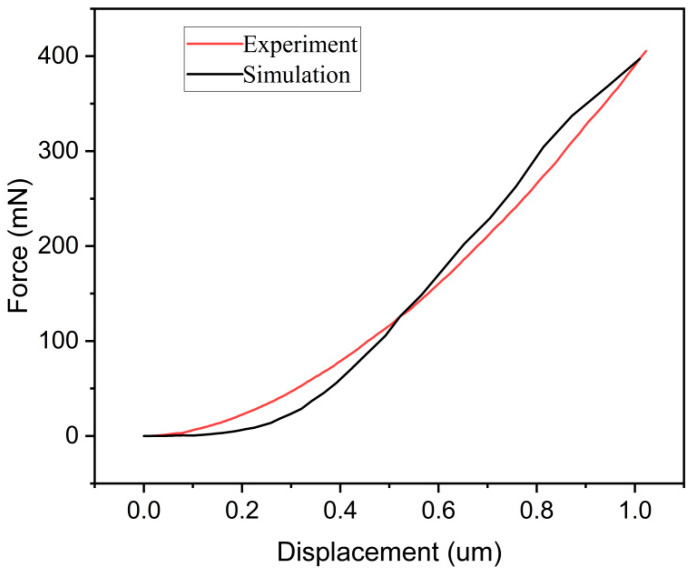
Simulated and experimental load–displacement curves.

The surface topography serves as a means to validate the accuracy of the simulation model’s results qualitatively. Meanwhile, the load–displacement curve provides a quantitative and intuitive representation of the dynamic material changes during the indentation experiment. The curve generally includes a loading stage and an unloading stage. However, due to the absence of unloading conditions in the simulation model, we have chosen to compare only the load–displacement curve of the loading phase. Figure 5 shows a comparative analysis of the experimental and simulated load–displacement curves. In the graph, it is evident that the growth trends of the load–displacement curves of the experiment and simulation are basically consistent, and the curve coincidence degree is high. There is a high degree of agreement between the experimental data and the simulation data, which proves that the indentation simulation model used in this article is highly accurate and can be used for subsequent indentation simulation.

## 3. Results and Discussion

In this paper, all the simulation analyses are performed in LS-DYNA R11 (Livermore Software Technology Corporation (LSTC), Troy, MI, USA). The simulation parameters, as shown in Table 4, were utilized to investigate the influence of pressure on the damage of 4H-SiC through indentation simulations. The aim was to obtain a low-damage machining method. The force parameters in Table 4 are selected based on Equation (13) as follows:

(13) Fn=P∗S/n

where the commonly used 6-inch wafer area *S* is approximately 182.3 cm^2^, with typical processing pressures *P* ranging from 20 to 50 kPa. The effective number of abrasive grains n during processing is about 8000. Then, the processing pressure per grain Fn can be calculated to a range of approximately 50 to 100 mN.

The results were processed using LS-PrePost (LSTC, USA). The analysis focused on examining the cracking behavior induced by different pressures. It also involved observing stress distribution on the surface and sub-surface of the 4H-SiC workpiece. The goal was to discuss the influence of pressure on stress distribution within the 4H-SiC workpiece and, based on the simulation results, establish a predictive model for the depth of indentation.

### 3.1. The Effect of Pressure on Depth

**Figure 6:**
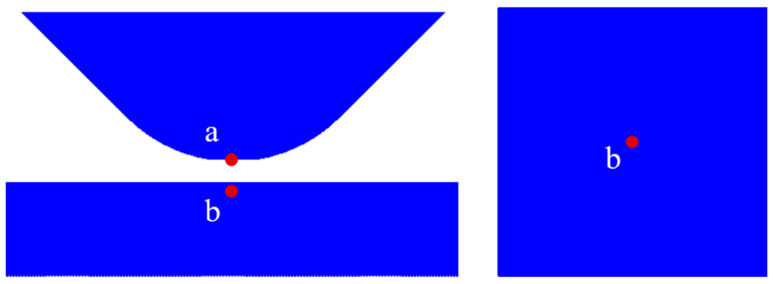
Measurement point labeling.

Due to the brittleness of 4H-SiC, it is prone to crack or fracture during indentation simulations. The occurrence of cracks or fractures in the material can result in displacement or splashing of the SPH particles. In order to ensure the effective reading of indentation depth changes and stress changes, this study defines the measurement points for the indentation depth at the tip of the diamond abrasive particles. The measurement points for stress changes are defined at positions corresponding to the center of the workpiece and the tip of the abrasive particle, as shown in Figure 6, denoted by points a and b, respectively.

**Figure 7:**
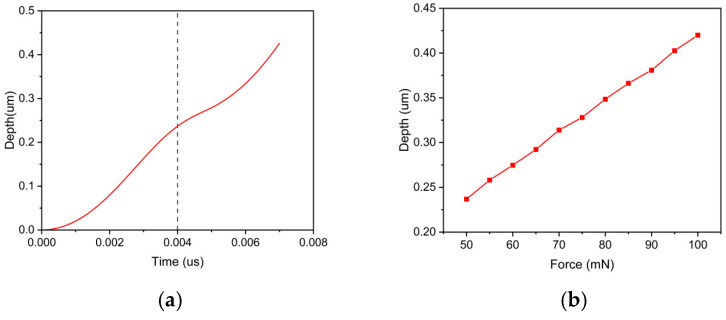
(**a**) Depth variation during the penetration process. (**b**) Transition depth at different pressures.

The removal process of brittle materials typically involves three scenarios: elastic deformation, plastic deformation, and brittle fracture [24,25]. Figure 7a shows the variation in the indentation depth with time under a 50 mN loading pressure. From the graph, it can be observed that under the 50 mN pressure condition, the indentation depth increases with time. The penetration velocity of the abrasive particle exhibits a trend of initial increase, followed by a decrease, and then another increase. The inflection point is near the dotted line in the graph, which conveniently corresponds to the three stages of brittle material removal. Because of the constant external load input, as the depth of abrasive particle penetration increases, the contact area between the abrasive particle and the workpiece also increases. Consequently, the effective stress decreases, leading to a reduction in the penetration velocity of the abrasive particle. When the abrasive particle reaches a certain penetration depth, the workpiece experiences fracture, causing a reversion in the penetration velocity. According to the expression of the JH-2 constitutive model [22], material damage will cause the strength of the material to decrease. At this point, the abrasive particle only requires a more minor effective stress to penetrate the workpiece. Therefore, as shown in Figure 7a, the curve continues to increase in the later stages. Under different loading pressures, the depth at which the downward pressure velocity changes is at different locations. Figure 7b shows the depths of these transition points at other pressures. The depth of the transition point deepens as the pressure increases.

The pressing depth of abrasive grains is affected by various factors such as loading pressure, abrasive grain hardness, and abrasive grain size. Among these factors, loading pressure is particularly crucial. If the pressure is too low, the abrasive particle may fail to penetrate the workpiece or achieve the desired depth. On the other hand, excessive pressure can lead to overly deep penetration, affecting the machining efficiency of the workpiece. Therefore, a mathematical model that describes the relationship between loading pressure and penetration depth through numerical simulation is essential for predicting the depth of abrasive particle penetration under specific pressures. Assuming a linear relationship between the pressure *F_n_* and the penetration depth *a_p_*, the expression for the relationship between pressure and penetration depth can be represented as follows:

(14) $$a_{p}=A*F_{n}+B$$

where *a_p_* is the penetration depth, *F_n_* is the loading force, and *A* and *B* are constants.

**Figure 8:**
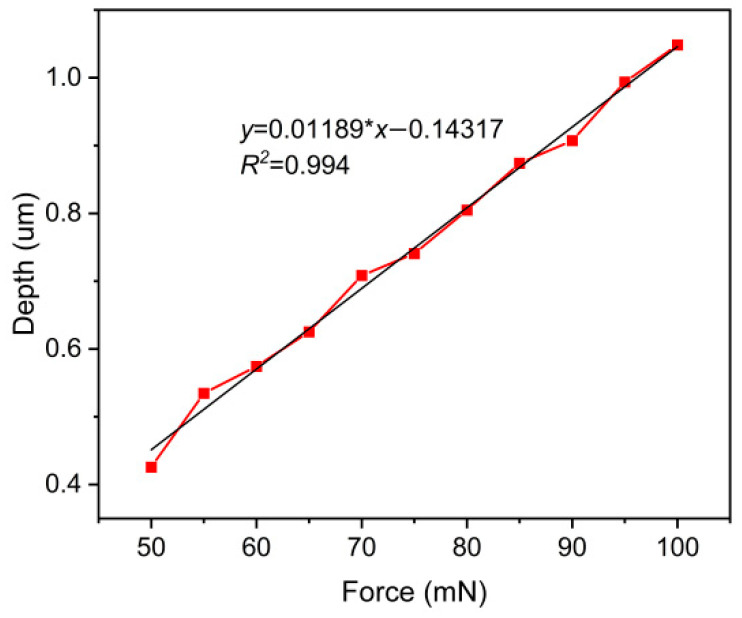
Penetration depth under different pressures.

The above simulation results were used to perform first-order polynomial fitting, yielding the results shown in Figure 8, where the constants *A* and *B* were determined to be 0.01189 and −0.14317, respectively. From the results in the graph, it can be observed that the penetration depth of abrasive particles increases linearly with the increase in pressure within the same simulated time. The relationship is an approximately linear correlation, with an R-squared value of 0.994, indicating a high degree of fitting accuracy. Therefore, fitting was used, as mentioned earlier, to predict the penetration depth of abrasive particles under a fixed load.

### 3.2. The Effect of Pressure on Stress Distribution

**Figure 9:**
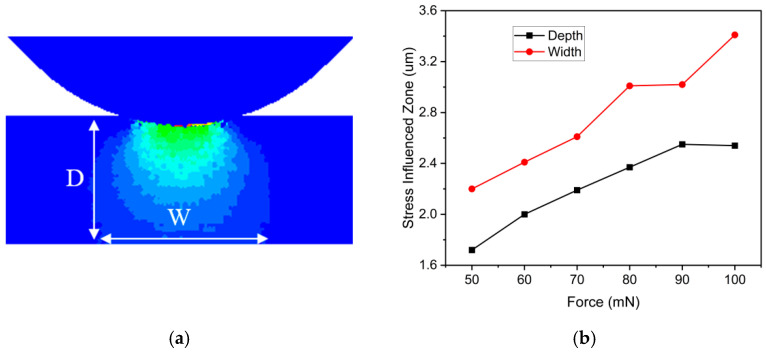
(**a**) Stress-affected zone. (**b**) Size of the stress-affected zone under different pressures.

During the indentation simulation, the workpiece experiences both tensile and compressive stresses. 4H-SiC, inherently brittle, exhibits significant differences in its mechanical properties under tension and compression. When the external load reaches the maximum tensile stress, the material fractures and fails, simultaneously releasing stress. However, in the simulations presented in this paper, the workpiece primarily undergoes compressive stress induced by the abrasive particles. Compressive stress, under certain conditions, can enhance the material’s strength. Before reaching its ultimate limit, compressive stress manifests on the workpiece in various forms, including plastic deformation and residual stresses. As depicted in Figure 9a, when abrasive particles make contact with the workpiece, they create a stress-affected zone. This zone is mainly concentrated directly beneath the abrasive particles and displays a sinusoidal distribution along the depth of the workpiece. A notable stress concentration is observed near the tip of the abrasive particles.

As the abrasive particles exert downward pressure, the resulting stress-affected zone extends further into the workpiece. This zone is affected not only by the material’s intrinsic characteristics but also by external loads. Evaluating the influence of changes in external loads on the stress-affected zone necessitates measurements of both depth and width, denoted as D and W in Figure 9a. Results from previous simulations are depicted in Figure 9b, demonstrating a clear trend of expansion in the stress-affected zone within the workpiece as external loads increase.

### 3.3. The Effect of Pressure on Crack Damage

**Figure 10:**
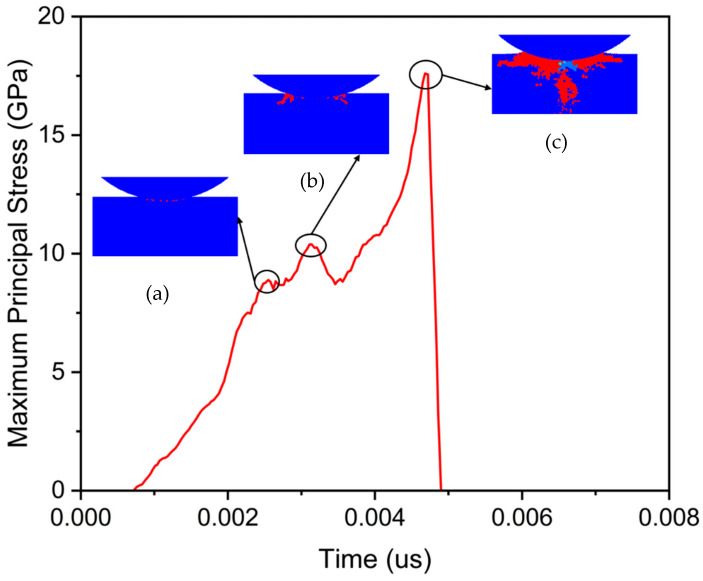
The stress variation at point b: (**a**) crack condition at 0.0025 μs; (**b**) crack condition at 0.0032 μs; (**c**) crack condition at 0.0047 μs.

The variation in stress is closely associated with the initiation and propagation of cracks, and the following analysis explores the relationship between stress changes and crack extension. As shown in Figure 6, point b within the workpiece was selected to study stress variation under a 50 mN loading pressure. The stress variation at point b is shown in Figure 10, where stress increases from the moment the abrasive particle contacts the workpiece. During the rising phase of the curve, there are several small fluctuations. After reaching its maximum value, the stress suddenly drops to zero, which is more in line with the typical stress behavior of brittle materials during the loading process.

The generation of cracks leads to the dissipation of stress, consequently causing sudden changes in stress levels. Examining the curve in Figure 10, it is evident that stress experienced three distinct and pronounced discontinuities during the ascent. These three stress discontinuity points are indicative of crack formation in the workpiece. The first stress discontinuity point occurs at 0.0025 μs, as shown in Figure 10a. At this moment, the workpiece exhibits a minor amount of cracking. The second stress discontinuity point is at a time of 0.0032 μs, as shown in Figure 10b. Here, the workpiece displays more pronounced cracks, predominantly in the transverse direction. The magnitude of stress change at this point is larger than at the previous one, indicating a greater extent of cracking. At 0.0047 μs, the stress suddenly drops from its maximum value to zero, as shown in Figure 10c. At this point, significant fragmentation of the workpiece occurs. During this fracture event, separation between the abrasive particles and the workpiece causes stress to reduce to zero.

Based on the above results, the emergence of cracks is attributed to the external loading reaching the damage threshold of 4H-SiC. The presence of cracks leads to the release of a portion of stress, and the more cracks develop, the greater the stress released. When the accumulated damage from cracks in the workpiece reaches a certain level, the workpiece experiences fracture.

## 4. Discussion

**Figure 11:**
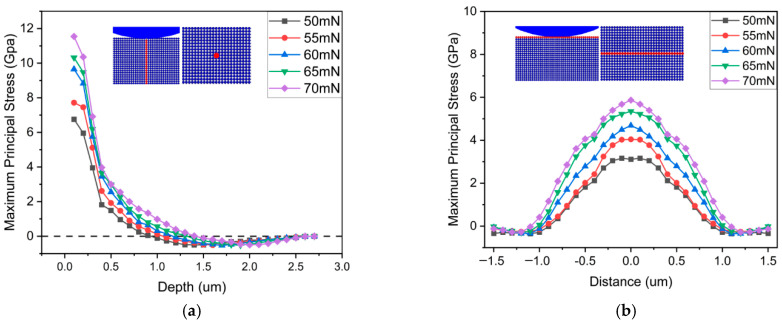
(**a**) Longitudinal stress distribution trend. (**b**) Transverse stress distribution trend.

Identifying trends in stress distribution during indentation is a dynamic process that necessitates further analysis. As shown in Figure 11a, the red-marked points denote stress variations at each specific point. The stress trends along the longitudinal axis of the workpiece are delineated by the curve in Figure 11a. In the initial segment of the curve, corresponding to the workpiece’s surface, stress increases with the applied load and experiences a rapid decline. This phenomenon is primarily attributed to the significant stress concentration directly beneath the abrasive particle. As the depth increases, stress diminishes, revealing an overall decreasing trend. Beyond 0.1 μm, the curve exhibits negative stress values, attributed to tensile stresses in that particular location.

Utilizing the same methodology, the transverse stress distribution trend of the workpiece was assessed. Originating from the central point where the abrasive particle contacts the workpiece, extensions were made to both the left and right sides, as indicated by the red-marked points in Figure 11b. The curve depicted in Figure 11b illustrates the transverse stress variation trend. Clearly observed from the graph is the symmetry of the curve about x = 0, with the peak situated in the middle section and gradually diminishing on both sides. As previously noted, a notable stress concentration exists directly beneath the abrasive particle. The farther one moves from the abrasive particle, the lesser the impact of stress. Due to the conical shape of the abrasive particle, the contact area between the abrasive particle and the workpiece remains relatively uniform during the indentation process, resulting in a comparatively even distribution of stress effects. This contributes to the observed trend in the graph, depicting a higher middle section and lower ends.

By analyzing the stress distribution trends under various loading pressures, we observed that the longitudinal and transverse stress variation trends exhibit remarkable similarities, albeit with variations in stress magnitude. Consequently, it becomes feasible to represent these trends using a mathematical model. In this study, we define the horizontal distance from each point to the center of contact between the abrasive particle and the workpiece as *d_x_*, and the vertical distance as *d_y_*. Building upon the simulation outcomes outlined previously, we can summarize the stress distribution pattern in the workpiece as follows:

(15) $$\begin{array}{ll} \sigma_{y}= & (-23.17-n*1.6)d_{y}+(20.3+n)d_{y}^{2}+(-7.5-n*0.5)d_{y}^{3}\\ & +(1+n*0.1)d_{y}^{4}+(0.18+n*0.01)F \end{array}$$

where *F* is the loading pressure; n is the different stress loading conditions, with *n* = 0, 1, 2, 3, 4; F=50+n∗5; the error in the coefficient of the $d_{y}$ term is ±0.5; and the error in the coefficient of the $d_{y}^{3}$ term is ±0.05.

Due to the symmetric nature of the transverse stress distribution curve, it is only necessary to consider the distribution pattern on one side. The distribution pattern is as follows:

(16) $$\begin{array}{ll} \sigma_{x}= & (2.6-n*0.2)d_{x}+(-19.5+n*1.5)d_{x}^{2}+(19-n*3.5)d_{x}^{3}\\ & +(-5.5+n*1.5)d_{x}^{4}+(0.0635+n*0.008)F \end{array}$$

where *F* and *n* are the same as in Equation (15); the error in the coefficient of the $d_{y}^{2}$ term is ±0.5.

In Equation (15), when the loading pressure *F* is fixed, *σ_y_* decreases overall with the increase in *d_y_*. The maximum value occurs at *d_y_* = 0.1 μm. Because of the influence of the magnitude of *F*, the extremum points of *σ_y_* exhibit fluctuation. The minimum value of σ_y_ appears near *d_y_* = 1.8 μm. In contrast, the situation in Equation (16) is different. *σ_x_* initially increases with the increase in *d_x_* and then decreases. The maximum value occurs at *d_x_* = 0.1 μm. Similarly, influenced by the magnitude of *F*, the points of minimum *σ_x_* also fluctuate, with an overall distribution around *d_x_* = 1.3 μm.

**Figure 12:**
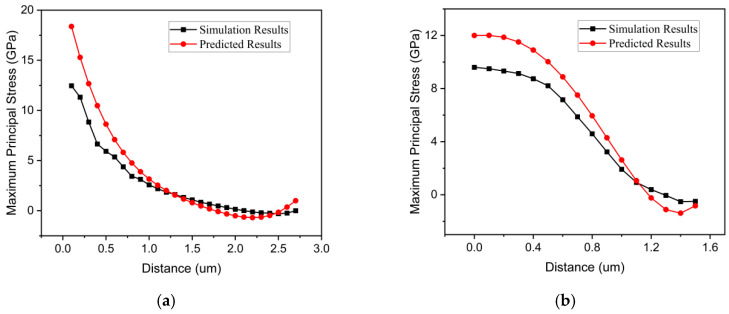
(**a**) Longitudinal distribution results. (**b**) Transverse distribution results.

Since 100 mN is the maximum pressure applied to a single abrasive grain during the grinding process, as long as the 4H-SiC does not undergo brittle failure under the maximum pressure—that is, the maximum equivalent stress is not zero—and the simulation results are basically consistent with the calculation results of Equations (15) and (16), the validity and accuracy of the equations can be ensured. Figure 12a,b show the simulation and prediction results under a 100 mN applied pressure. It can be observed from the figures that the predicted results closely align with the simulation results, following a similar trend. However, there is a noticeable deviation in the initial part of the curves. This discrepancy is primarily attributed to the fact that the front-end of the curve corresponds to the primary contact area between the abrasive particles and the workpiece, where significant stress concentration occurs. Additionally, cracks in the workpiece tend to initiate and propagate in this region, leading to some deviation in the prediction results.

## 5. Conclusions

This paper provides simulations of 4H-SiC indentation under varying loading pressures of 50–100 mN utilizing the SPH method. The results have been rigorously validated through experimental tests to confirm their reliability. The study elucidates the stress fluctuations in 4H-SiC material at different loading pressures and investigates the influence of pressure on indentation depth, stress alterations, and material damage. The following conclusions have been derived:

- The depth of abrasive particle penetration and the size of the stress-affected zone increase as the applied pressure grows, and the depth of abrasive particle penetration is approximately linearly related to the applied pressure magnitude.
- Significant stress concentration is present directly beneath the abrasive particles, with stress decreasing in magnitude in the direction moving away from the particles. There is a discernible pattern in the distribution of stress within the workpiece.
- Through simulation research, critical coefficients for the mathematical model of stress distribution within the workpiece have been obtained, and the accuracy of this mathematical model has been verified. It is capable of predicting the stress distribution patterns under varying applied pressures.

## Figures and Tables

**Table 1:** The constitutive parameters of diamond.

Parameter	Density *ρ* [kg/m^3^]	Young’s Modulus *E* [GPa]	Poisson’s Ratio [GPa]
Value	3500	964	0.07

**Table 2:** The constitutive parameters of SiC [23].

Parameter	Density *ρ* [kg/m^3^]	Young’s Modulus *E* [GPa]	Shear Modulus *G* [GPa]	Fracture Strength [GPa]
Value	3163	450	183	0.8
Parameter	*A*	*B*	*C*	*M*
Value	0.96	0.35	0	1.0
Parameter	*N*	EPSI	*T* [GPa]	HEL [GPa]
Value	0.65	1.0	0.37	14.457
Parameter	*P*_HEL_ [GPa]	BETA	*D*_1_	*D*_2_
Value	5.9	1.0	0.48	0.48
Parameter	*K*_1_ [GPa]	*K*_2_ [GPa]	*K*_3_ [GPa]	Poisson’s ratio
Value	204.8	0	0	0.16

**Table 3:** Experimental and simulation parameters.

Parameter	Value	
	Experiment	Simulation
Type	Berkovich	Berkovich
Workpiece size (mm)	10 × 10	0.015 × 0.015
Depth (μm)	1	1

**Table 4:** Indentation simulation parameters.

Simulation Parameters	Value
Force (mN)	50, 55, 60, 65, 70, 75, 80, 85, 90, 95, 100
Termination (μs)	0.007

## Data Availability

The raw data supporting the conclusions of this article will be made available by the authors on request.
